# The Potential of Alternative Therapies and Vaccine Candidates against *Helicobacter pylori*

**DOI:** 10.3390/ph16040552

**Published:** 2023-04-06

**Authors:** Asif Sukri, Alfizah Hanafiah, Sandip Patil, Bruno S. Lopes

**Affiliations:** 1 Department of Biological Sciences and Biotechnology, Faculty of Science and Technology, Universiti Kebangsaan Malaysia, Bangi 43600, Selangor, Malaysia; 2 Department of Medical Microbiology and Immunology, Faculty of Medicine, Universiti Kebangsaan Malaysia, Kuala Lumpur 56000, Malaysia; 3 Department of Hematology and Oncology, Shenzhen Children’s Hospital, Shenzhen 518038, China; 4 School of Health and Life Sciences, Teesside University, Middlesbrough TS1 3BA, UK; 5 National Horizons Centre, Teesside University, Darlington DL1 1HG, UK

**Keywords:** *Helicobacter pylori*, alternative therapy, plant, probiotic, nanoparticle, vaccine

## Abstract

Alternative therapies and vaccination are essential to combat the emergence of multidrug-resistant *Helicobacter pylori* and to prevent the development of gastroduodenal diseases. This review aimed to systematically review recent studies on alternative therapies, i.e., probiotics, nanoparticles, and natural products from plants, as well as recent progress in *H. pylori* vaccines at the preclinical stage. Articles published from January 2018 to August 2022 were systematically searched using PubMed, Scopus, Web of Science, and Medline. After the screening process, 45 articles were eligible for inclusion in this review. Probiotics (*n* = 9 studies) and natural products from plants (*n* = 28 studies) were observed to inhibit the growth of *H. pylori*, improve immune response, reduce inflammation, and reduce the pathogenic effects of *H. pylori* virulence factors. Natural products from plants also showed anti-biofilm activity against *H. pylori*. However, clinical trials of natural products from plants and probiotics are still lacking. A paucity of data assessing the nanoparticle activity of N-acylhomoserine lactonase-stabilized silver against *H. pylori* was observed. Nonetheless, one nanoparticle study showed anti-biofilm activity against *H. pylori*. Promising results of *H. pylori* vaccine candidates (*n* = 7) were observed at preclinical stage, including elicitation of a humoral and mucosal immune response. Furthermore, the application of new vaccine technology including multi-epitope and vector-based vaccines using bacteria was investigated at the preclinical stage. Taken together, probiotics, natural products from plants, and nanoparticles exhibited antibacterial activity against *H. pylori*. New vaccine technology shows promising results against *H. pylori*.

## 1. Introduction

*Helicobacter pylori* infects more than 50% of the world’s population and causes gastroduodenal diseases including gastritis, peptic ulcer, gastric adenocarcinoma, and gastric lymphoma; it has been classified as a type I carcinogen that causes gastric cancer [1]. Gastric cancer is still one of the leading causes of cancer-related death worldwide although the prevalence and incidence of this cancer have been decreasing since the last decade [2]. Eradication of *H. pylori* is recommended to prevent gastric cancer, especially in developing countries where gastric cancer contributes to high economic morbidity and mortality [3]. Treatment of *H. pylori* includes administration of multiple antibiotics, namely, clarithromycin, amoxicillin, metronidazole, and tetracycline [4]. However, the emergence of *H. pylori* strains that are resistant to multiple antibiotics has complicated the treatment strategy to eradicate this bacterium [5]. In 2018, the World Health Organization listed *H. pylori* as one of the high-priority pathogens for research and discovery of novel drugs [6].

Multiple alternative therapies, including natural products, probiotics, and nanoparticles, can be assessed for antibacterial activity against *H. pylori*. A previous systematic review explored the efficacy of antimicrobial peptides natural sources as a promising alternative therapy for *H. pylori* [7]. Numerous natural products from plants have also been demonstrated to possess antibacterial activity against *H. pylori*, and they have been used as traditional medicine in some cultures such as East Asian and Southeast Asian cultures to treat multiple infections [8]. Probiotics are generally regarded as safe microbes that have been shown to give benefits to humans and are usually isolated from the fermentation process, including traditional foods [9]. In addition to probiotics and natural products from plants, applications of nanoparticles in drug delivery for antibiotics, implantable medical devices, and bone cement have also been explored for antibacterial effects against multiple bacteria [10]. Despite evidence demonstrating the efficacy of natural products from plants and probiotics against *H. pylori*, they are still not widely approved for therapy of *H. pylori*. Prevention of *H. pylori* infection through vaccination is also pertinent to prevent gastroduodenal diseases. To date, no vaccine for *H. pylori* has been approved. The objective of this review was to systematically review recent studies on alternative therapies (natural products from plants, probiotics, and nanoparticles) against *H. pylori* and progress of *H. pylori* vaccines.

## 2. Results

### 2.1. Literature Assessment

**Figure 1:**
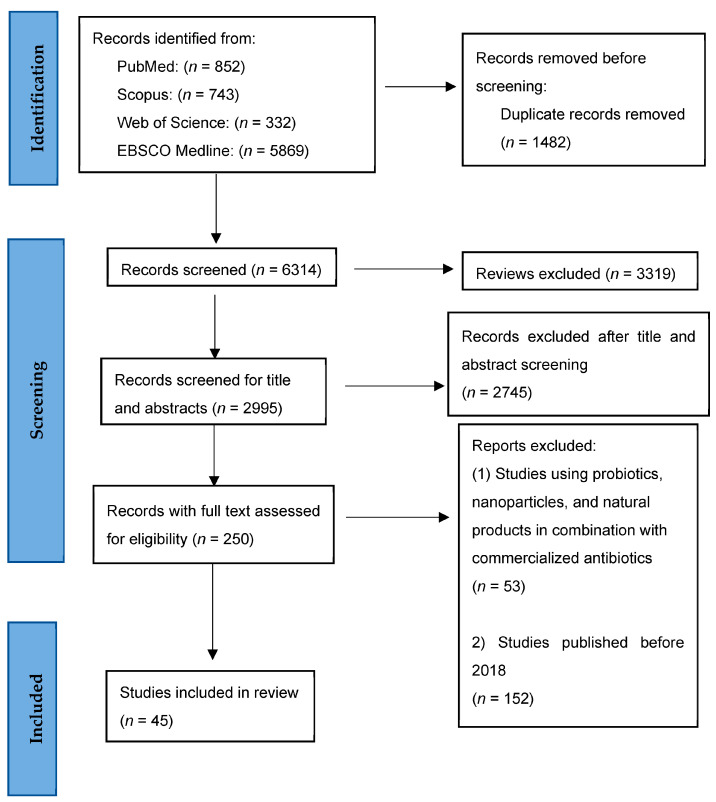
Flow diagram outlining article screening for inclusion in this systematic review using PRISMA guidelines. After screening evaluation, 45 articles were eligible for inclusion in this systematic review.

Figure 1 illustrates the screening process adopted for inclusion and exclusion of the articles in this systematic review. A total of 7796 articles were obtained from the following literature databases: PubMed (*n* = 852), Scopus (*n* = 743), Web of Science (*n* = 332), and EBSCO Medline (*n* = 5869). After we removed the duplicates (*n* = 1482) and reviews (*n* = 3319) using Microsoft Excel 2016 and Mendeley reference manager, 2995 articles were eligible for title and abstract screening. An additional 2745 articles were excluded because they were not relevant to the research question. Hence, 250 articles were eligible for full-text evaluation, of which 205 articles were excluded because the studies (*n* = 53) used natural products, probiotics, or nanoparticles in combination with commercial antibiotics, and the studies were published prior to 2018 (*n* = 152). Finally, 45 articles were eligible to be included in this systematic review. Overall, seven studies (15.5%) were published in 2018, along with 10 (22.2%) in 2019, 10 (22.2%) in 2020, 10 (22.2%) in 2021, and eight (17.8%) in 2022. Most of the studies published were preclinical studies (*n* = 44; 97.8%), while only one study (2.2%) was a clinical trial. Nine studies evaluated potential probiotics against *H. pylori*, 28 studies evaluated plant natural products against *H. pylori*, one study evaluated nanoparticles only, and seven studies evaluated vaccine candidates.

### 2.2. Summary of Studies on Alternative Therapies and Vaccines Conducted Prior to 2018

As the objective of our review was to systematically review recent studies published on alternative therapies and vaccines against *H. pylori* from 2018 to 2022, we excluded studies published prior to 2018. However, we briefly summarize breakthrough discoveries on this topic before 2018. According to our search, numerous studies on alternative therapies against *H. pylori* were published prior to 2018. Most of the studies examined the antibacterial activity of products from plants against *H. pylori* with promising results, including inhibition of growth in vitro, bacterial load reduction in animal models, and suppression of *H. pylori* virulence factors. Additionally, most studies were conducted at the preclinical level. Progress has been made in the discovery of vaccines against *H. pylori*, including a clinical trial conducted in China (NCT02302170), where they found the administration of oral recombinant *H. pylori* in children to be safe and effective in preventing *H. pylori* infection [11]. In probiotics research, one clinical trial using a combination of eight bacteria administered to 40 patients showed promising results, whereby *H. pylori* was eradicated in 13 patients [12]. In summary, we found potential clinical application of alternative therapies and vaccines against *H. pylori* before 2018.

### 2.3. Antibacterial Activity of Probiotics against H. pylori

Nine studies assessed the antibacterial activity of potential probiotics against *H. pylori*, of which eight were preclinical studies while one was a clinical trial (Table 1).

Most studies assessed the antibacterial activity of *Lactobacillus* spp. (8/9; 88.9%) against *H. pylori*, of which four studies determined the antibacterial properties of *L. acidophilus*, two studies determined the properties of *Bifidobacterium* spp., and one study each determined the properties of *Streptococcus thermophilus* and *Parabacteroides goldsteinii* MTS01. Of note, *Lactobacillus* spp. were demonstrated to show antibacterial activity against *H. pylori* in vitro and in vivo studies. Four studies showed that *Lactobacillus* spp., namely, *L. casei*, *L. paracasei*, *L. acidophilus*, *L. rhamnosus*, and *L. fermentum*, inhibited the growth of *H. pylori* in vitro. Notably, one study showed that *L. rhamnosus* and *L. acidophilus* inhibited the growth of multidrug-resistant *H. pylori* in vitro [14] (Table 1). *S. thermophilus* and *B. lactis* also inhibited the growth of *H. pylori*. Synthesis of data based on the antibacterial mechanism of probiotics against *H. pylori* revealed that most studies (*n* = 4) found the administration of probiotics in either animal models or humans altered the gut microbiota of the host infected with *H. pylori*. In one study, mice infected with *H. pylori*, but treated with probiotics, were shown to harbor enriched beneficial microbes that produced short fatty acid chains such as *Bacteroides*, *Alloprevotella*, and *Oscellibacter* and anti-inflammatory microbes (*Faecalibaculum*) [20]. However, in a clinical trial, probiotic monotherapy consisting of *B. infantis*, *L. acidophilus*, *E. faecalis* (>0.5 × 10^6^ CFU/tablet), and *B. cereus* for 14 days was not beneficial to *H. pylori*-infected subjects as the therapy was not successful in reducing *H. pylori* burden in human’s stomach (ChiCTR1900024893) [15]. Three studies found that administration of probiotics reduced inflammation in the host, in which the expression of interleukin-8 (IL-8) and nuclear factor kappa B (NF-κB) decreased in *H. pylori*-infected cell lines or animal models. In addition, molecules essential in proinflammatory cellular signaling were reduced in the *H. pylori*-infected host model [20]. Administration of probiotics also reduced *H. pylori* cell adhesion, as observed in two studies [14,16]. An increase in cell apoptosis was observed in two studies [17,19]. Furthermore, probiotics treatment was also demonstrated to reduce the effect of vacuolating cytotoxin A (VacA) vacuolation in cells [18,21], as well as cytotoxin-associated gene A (CagA) translocation, phosphorylation, and the “hummingbird” cell-scattering effect [14,21]. Lin et al. [18] also found that treatment of probiotics reduced *H. pylori* colonization burden and stimulated the release of metabolites important in the immune response in mice.

### 2.4. Antibacterial Activity of Natural Products from Plants and Nanoparticles against H. pylori

Supplementary Table S1 [22,23,24,25,26,27,28,29,30,31,32,33,34,35,36,37,38,39,40,41,42,43,44,45,46,47,48,49] shows a list of plants used to examine the antibacterial activity of natural products from plants against *H. pylori*. In total, 28 studies examining the antibacterial activity of natural products against *H. pylori* were included in this review. Most studies (*n* = 15) assessed the antibacterial activity of plant extracts, while the remaining studies (*n* = 13) assessed isolated compounds from plants. The minimal inhibitory concentration (MIC) value was assessed in 20 studies, among which eight studies also assessed the minimal bactericidal concentration (MBC) value. Most studies that reported the MIC value (*n* = 16) adopted the broth microdilution assay to assess MIC value, whereas the remaining studies used the disc diffusion assay (*n* = 2) or both the disc diffusion assay and the agar dilution method (*n* = 2). Nineteen out of 20 studies (95%) providing the MIC value reported the susceptibility of *H. pylori* against the plant extracts or isolated compounds examined, while one study (1/20; 5%) reported no direct antibacterial activity. The lowest MIC value was 1.25–5 µg/mL in a study that examined the compound nimbolide isolated from *Azadirachta indica* [41]. In addition, this compound from a similar study also demonstrated an MBC against *H. pylori* ranging from 2.5 to 10 μg/mL. A high MIC value ≥500 µg/mL was reported in the studies that examined the antibacterial activity of the compound taxifolin from *Mimusops balata* fruit [25], the ethyl acetate fraction of *Physalis alkekengi* L. var. *franchetii*, and the dry extract of *Libidibia ferrea*. The antibacterial mechanisms of plant extracts or compounds were examined in 20 studies.

Overall, 10 categories of antibacterial mechanism were examined in the studies: (1) anti-biofilm, (2) anti-urease, (3) gastroprotection, (4) anti-inflammation, (5) effect on *H. pylori* virulence factors, (6) ATP leakage from *H. pylori*, (7) immune response, (8) *H. pylori* conversion from spiral to coccoid (inactive form), (9) cellular signaling, and (10) bacterial burden. Two studies found that the ethyl acetate fraction from *Hibiscus rosa-sinensis* red flower [29] and phylligenin, a compound isolated from flowering plant *Forsythia* [37], exhibited anti-biofilm activity against *H. pylori*. Meanwhile, four studies found that natural plant products possessed anti-urease activity, and six studies showed the gastroprotective properties of plant natural products. Furthermore, six studies demonstrated the anti-inflammatory activity of six different plant products. Five studies showed a reduction in *H. pylori* virulence factors, namely, *cagA*, *vacA*, *ureA*, *flaA*, and Omp18, while three studies showed a reduction in bacterial load and colonization. Two studies each demonstrated a reduction in cellular signaling important in *H. pylori*-induced carcinogenesis [30,48] and conversion of *H. pylori* from the active (spiral) to inactive coccoid form [29,42]. Li et al. [37] observed that phylligenin extracted from *Forsythia* induced ATP leakage in *H. pylori* and inhibited the mechanism of antibacterial resistance in the bacteria, as well as induced a good immune response.

It is suggested that to be considered as good antibacterial agent against *H. pylori*, the potential therapeutic candidate natural product must have antibacterial activity against not only the bacteria itself, but also its virulence factors that orchestrate gastric carcinogenesis. Taken together, the studies included in this systematic review (Table S1) demonstrated the application of natural products that can be used as future alternative therapies against *H. pylori*. Mechanisms targeting *H. pylori* examined in this study included the effects of the products from plants against important *H. pylori* virulence factors, namely, urease and CagA, which are both important for *H. pylori* to establish colonization in the human stomach due to its inhospitable environment. Furthermore, the effect of plant compounds in decreasing CagA activity is also important to prevent gastric carcinogenesis orchestrated by this oncoprotein [30,48]. Nevertheless, modeling of the natural products against specific target molecules of *H. pylori* is still lacking. The interaction of potential compounds with *H. pylori* using molecular docking and machine learning, coupled with in vitro and in vivo experiments, should be conducted in the future to elucidate the mechanism of antibacterial activity.

Nanoparticles have emerged alternative antibacterial agents against *H. pylori* because of their chemical properties that enable the attachment to and disturbance of the membrane, targeting bacterial DNA replication and transcription, as well as RNA translation [50]. In this review, only one study [51] examined the antibacterial activity of nanoparticles against *H. pylori*. The nanoparticles (namely, N-acylhomoserine lactonase-stabilized silver nanoparticles (AiiA-AgNPs)) inhibited the quorum sensing molecules of *H. pylori*, which in turn reduced the biofilm formation of the bacteria, production of urease, and cell surface hydrophobicity of *H. pylori* [51].

### 2.5. Progress on H. pylori Vaccine

Seven studies that evaluated vaccine candidates against *H. pylori* were included in this systematic review. A list of the vaccine candidates examined is available in Table 2.

All studies adopted mouse models to study the vaccine efficacy. The types of vaccine adopted in the studies included vector-based vaccines using vectors of *Saccharomyces cerevisiae* (*n* = 1), *Listeria monocytogenes* (*n* = 1), and *Lactococcus lactis* (*n* = 1). Multivalent epitope vaccines were employed in two studies, and outer membrane vesicles and peptide antigens were used in one study each. Six studies (85.7%) administered vaccine delivery to the animal models orally, while one study (14.3%) administered the vaccine through subcutaneous injection. *H. pylori* virulence factors targeted for vaccine design included urease, CagA, VacA, FlaA, neutrophil-activating protein A subunit, outer membrane vesicles, AlpB, SabA, and HpaA. Two studies that employed multivalent epitope vaccines targeted epitopes on B and T cells. A study conducted by Liu et al. [55] targeted 169 outer membrane proteins of *H. pylori*. In summary, all vaccine candidates showed promising results at a preclinical level. All studies observed higher IgA and IgG levels in mice immunized with vaccine candidates compared to controls. A reduction in the bacterial load and stomach inflammation were observed in most studies (6/7; 85.7%). Furthermore, three studies showed that immunization elicited an immune response in mouse models, including an increase in the secretion of interferon gamma (IFN-γ), IL-4, and IL-17. Contradictory T-cell polarization was observed, whereby one study [55] observed T helper 2 (Th2) polarization in immunized mice while Peng et al. [56] observed Th17 and Th1 polarization.

## 3. Discussion

In this systematic review, we systematically reviewed the antibacterial activity of probiotics, nanoparticles, and natural products from plants against *H. pylori*, as well as progress in *H. pylori* vaccine development. We restricted our analysis to articles published from 2018 to 2022 to obtain insights into recent publications describing alternative therapies against *H. pylori*. Studies that assessed the antibacterial activity of alternative therapies in combination with approved commercialized antibiotics were excluded from our review because the objective of our review was to assess the efficacy of alternative therapy administered as monotherapy. This is because antibiotics have been shown to disturb the gut microbiota and microbiome, which can have negative consequences for gastrointestinal health [59]. Additionally, antibiotic treatment regimens to eradicate *H. pylori* consist of multiple antibiotics, which may hinder patient compliance and contribute to an increase in the *H. pylori* antibiotic resistance rate. Hence, the discovery of alternative monotherapy is pertinent in the research and development of new drugs against *H. pylori*. Nevertheless, studies have shown that supplementation of probiotics together with antibiotics has helped to eradicate *H. pylori* infection in patients [60,61]. Despite an observed increase in publications from 2018 to 2022 regarding the alternative therapy of *H. pylori* in preclinical studies, a lack of publications on human studies was noted. Factors that contribute to a lack of clinical trials on potential therapies include a lack of financial resources and skilled medical specialists, as well as government regulations and administrative issues, specifically in middle- and low-income countries [62]. As the prevalence of *H. pylori* is high in middle- and low-income countries [63], collaboration between high-income countries and middle- and low-income countries in clinical trials should be encouraged to mitigate this issue. The studies included in this systematic review showed that some probiotics exhibited antibacterial activity or competitive exclusion against *H. pylori*. Furthermore, antibacterial mechanisms of probiotics against *H. pylori*, including effects on virulence factors, the gut microbiota, and the immune response, were also explored. However, there was a lack of studies that identified and assessed the antibacterial activity of compounds secreted by probiotics against *H. pylori*. This gap should be examined in future studies. Of note, the clinical trial included in this review that investigated the effect of probiotic monotherapy on human subjects failed to show a beneficial effect of probiotics in human subjects. In contrast, administration of probiotics together with antibiotics was demonstrated to improve eradication with minimal adverse effects [64]. Thus, the mechanism (i.e., drug synergism and antagonism of probiotic monotherapy and probiotic supplementation with antibiotics) of combating *H. pylori* infection in human should be further investigated in the future.

A promising application of natural products from plants was also observed in this systematic review. Every study adopted different plant species to determine antibacterial activity, suggesting a broad spectrum of plant types that can be used as therapy against the bacterium. The antibacterial activity of plant species against *H. pylori* has mostly been determined from plant extracts without an identification of specific compounds with activity against *H. pylori*. Given the complexity of plant chemical constituents, isolation of desired chemical compound from plants is important to determine the safety and the antibacterial efficacy of the compound intended for future human study. Most studies reported MIC values of the products examined. Nevertheless, a lack of reports including MBC values was noted. MIC values only provide information on the growth inhibition of *H. pylori*, while MBC value provide information on bacteria killing. Studies to determine antibacterial activity in the future should also report the MBC value of *H. pylori*. The Clinical and Laboratory Standard Institute (CLSI) recommends the agar dilution method to determine the MIC value of *H. pylori* [65]. However, we observed that most studies employed the broth microdilution assay to determine the MIC value. This discrepancy is because the agar dilution assay is difficult to perform and laborious, in addition to the fact that *H. pylori* grows slowly. Various antibacterial mechanisms of plant products against *H. pylori* were examined, including effects on virulence factors, bacterial burden in the host, and anti-biofilm activity. Although the emergence of multidrug-resistant *H. pylori* has been observed in Southeast Asia [7], there is a paucity of recent studies examining the antibacterial activity of plant natural products in this region. Given the richness of tropical biodiversity in this region, the anti-*H. pylori* activities of products from plants should be explored in the future using the diverse plant species in the region. Nanoparticles have been demonstrated to display antimicrobial activity against multiple types of bacteria [66]. Nevertheless, only one recent study assessed the antibacterial activity of nanoparticles against *H. pylori* with promising results. Barriers to the application of nanoparticles as antibacterial agents include the design of nanoparticles for efficient delivery to the host and the toxicity of the chemical to the host [67].

Vaccination remains a strategy to prevent infection. To date, no approved *H. pylori* vaccine is available. Nevertheless, phase III clinical trials of an *H. pylori* oral vaccine in 4464 participants were conducted with effective, safe, and immunogenic results [11]. All studies conducted at the preclinical stage in this systematic review demonstrated the promising results of *H. pylori* vaccine candidates, in which the host’s immune response was elicited in all studies. Interestingly, most studies explored the application of new vaccine technologies including vector-based vaccines using bacteria and multi-epitope vaccines targeting specific T and B cells. The unique design of multi-epitope vaccines harnesses a better immune response than single-epitope vaccines, particularly for *H. pylori* strains with different antigen variability [68]. Meanwhile, vector-based vaccines provide a robust immune response since live bacteria are easily recognized by the immune system, and since the delivery of live bacteria is more efficient compared to traditional vaccines [69]. Contradictory results regarding T-cell polarization have been found, whereby Peng et al. [56] found Th1 to be polarized in *H. pylori* vaccination, while Liu et al. [55] found Th2 to be polarized. Th2 cells are important to neutralize extracellular bacteria such as *H. pylori*, while Th1 cells are important to neutralize intracellular bacteria [70]. However, both studies showed promising results of *H. pylori* vaccine immunization. Further studies should be conducted to elucidate the importance of Th1 and Th2 cells in *H. pylori* immunization.

While the data obtained from the studies included in this systematic review that examined the alternative therapies against *H. pylori* are encouraging, there were several limitations in this review. Firstly, we noticed heterogeneity of the studies included in this systematic review. The methods adopted to examine antibacterial activity were not universal, whereby some studies used broth microdilution while other studies used an agar diffusion assay. However, the difficulty in culturing *H. pylori* in the laboratory with different types of plants contributed to the choice of selecting appropriate antibacterial assays that differ from one laboratory to another laboratory. Secondly, we only included studies that were published in English, which may have missed studies that were published in other languages. Lastly, most studies included in this review involved preclinical trials, thus hampering our understanding of whether the findings from preclinical studies can be translated to clinical trials. The lack of studies conducted on humans may have stemmed from the expensive cost to conduct clinical trials, especially in developing countries with limited funding resources and experts in respective fields. In addition, a lack of financial resources in developing countries is also one of the factors leading to a lack of product patents in preclinical studies that can be used for clinical trials before commercialization. Lack of expertise in managing clinical trials is also a challenge that developing countries must face before the implementation of clinical trials. Hence, this factor has contributed to a lack of clinical trials conducted in developing countries where *H. pylori* infection is high.

## 4. Materials and Methods

### 4.1. Literature Search

Four literature databases, namely, PubMed, Scopus, Web of Science, and EBSCO Medline, were used for literature search. We systematically evaluated all articles obtained from the literature search using Preferred Reporting Items for Systematic Reviews and Meta-Analyses (PRISMA) guidelines. The following keywords were used to search the articles: ((“natural product” OR “plant” OR “nanoparticle” OR “probiotic” OR “vaccine”) AND (“treatment”) AND (“*Helicobacter pylori*” OR “*H. pylori*”)). We defined natural products as compounds or extracts obtained from plant sources because our systematic review focused on natural products from plants. For probiotics, we defined the term as live microbes generally regarded as safe and administered to hosts that may have benefits for the hosts. Commercialized antibiotics are defined as antibiotics currently available to treat *H. pylori*, which include amoxicillin, tetracycline, clarithromycin, metronidazole, levofloxacin and rifampicin. Nanoparticles are ultrasmall particles in the range of 1–100 nm in diameter, while vaccines are defined as substances that can elicit an immune system response against *H. pylori*.

### 4.2. Inclusion and Exclusion Criteria

Inclusion criteria of this study included (1) studies that evaluated natural products from plants or nanoparticles against *H. pylori*, (2) studies that evaluated the antibacterial properties of potential probiotics against *H. pylori*, (3) studies that evaluated vaccine candidates of *H. pylori* in preclinical trials, (4) studies that were published in the last 5 years (from January 2018 to August 2022), (5) studies conducted in vitro, in vivo, or in humans, and (6) studies published in English with the full text available. Exclusion criteria included (1) studies that evaluated natural products, potential probiotics, or nanoparticles in combination with commercialized antibiotics against *H. pylori*, (2) studies that evaluated potential probiotics, natural products, vaccine, or nanoparticles against bacteria other than *H. pylori*, (3) studies not published in English or with full text not available, (4) studies that examined vaccine candidates in clinical trials, (5) book chapters, conference abstracts, and literature, systematic, or meta-analysis reviews, and (6) studies published before 2018. Two independent researchers evaluated the articles to be included in this review; if there was disagreement for inclusion or exclusion of the articles, a third researcher was consulted to reach consensus. The literature search was conducted from May to August 2022.

### 4.3. Data Extraction and Synthesis

Data such as authors, year, type of study (preclinical or clinical trials), type of natural products used in the study, name of probiotic or name of vaccine candidate and their evaluation in vitro and in vivo, and results of antibacterial activity against *H. pylori* were extracted and organized using Microsoft Excel 2016. Data were synthesized according to antibacterial activity and mechanism of the products against *H. pylori*.

## 5. Conclusions

In conclusion, probiotics and natural products from plants show promising results to be harnessed as alternative therapies against *H. pylori* to combat the emergence of multidrug-resistant strains. However, there is still a paucity of clinical trials on probiotics and natural products from plants. This stems from the fact that *H. pylori* infections are mostly diagnosed in developing countries where clinical trials are expensive and require a team of clinical experts. Consequently, the findings from preclinical studies cannot be properly translated to human studies. With the advent of artificial intelligence and machine learning technology, the research and development of drugs and vaccines are no longer limited to wet laboratory experiments; they can also include silico studies. Of note, studies that employed both wet and dry laboratory experiments for drug discovery were still lacking in our systematic review. In silico studies prior to commencement of wet laboratory experiments can provide valuable insights into potential drug candidates that can be screened in vitro and in vivo. Probiotics from fermented foods and natural products extracted from plants, especially from areas with rich biodiversity, can be harnessed for the research and discovery of novel antimicrobial agents against antibiotic-resistant *H. pylori*. *H. pylori* vaccines using new vaccine technologies show promising results in preclinical trials and should be explored further.

## Figures and Tables

**Table 1:** List of potential probiotics and their antibacterial activity against *H. pylori* included in this systematic review. UBT: urea breath test; LAB: lactic acid bacteria.

Authors	Type of Study	Name of Bacteria (Probiotics)	Results
Saracino et al. (2020) [13]	Preclinical	*L. casei*, *L. paracasei, L. acidophilus*, *B. lactis*, and *S. thermophilus*	Growth inhibition of *H. pylori*.
Chen et al. (2019) [14]	Preclinical	*L. rhamnosus* and *L. acidophilus*	Inhibited growth, adhesion, and invasion of *H. pylori*; reduced *H. pylori*-induced inflammation (decreased NF-κB activity and IL-8 secretion); downregulated phosphorylation and translocation of CagA; reshaped gut microbiota.
Yuan et al. (2021) [15]	Clinical	Probiotics therapy (*Bifidobacterium* tetravaccine tablets) included *B. infantis* > 0.5 × 10^6^ CFU/tablet, *L. acidophilus* > 0.5 × 10^6^ CFU/tablet, *E. faecalis* > 0.5 × 10^6^ CFU/tablet, *B. cereus* > 0.5 × 10^5^ CFU/tablet)	Upregulated pathogenic bacteria in gut microbiota after administration of probiotics.
Taghizadeh et al. (2020) [16]	Preclinical	*L. acidophilus* ATCC4356 and *L. rhamnosus* PTCC1607	Inhibited bacterial growth and adhesion; stimulated IFN-G.
Yarmohammadi et al. (2021) [17]	Preclinical	*L. gasseri* ATCC 33323	Downregulated the expression of IL-8 and Bcl2.
Lin et al. (2020) [18]	Preclinical	*L. fermentum* P2 (P2), *L. casei* L21 (L21), *L. rhamnosus* JB3 (JB3), or a mixture including the aforementioned three (multi-LAB) for 3 days	Modulated metabolites important in immune response.
Maleki-Kakelar et al. (2020) [19]	Preclinical	*L. plantarum*	Increased cell apoptosis.
He et al. (2022) [20]	Preclinical	*L. salivarius* and *L. rhamnosus*	Anti-inflammation (downregulated proinflammatory signaling pathways that included NF-κB, TNF, and IL-17; increased the abundance of beneficial bacteria in gut microbiota.
Lai et al. (2022) [21]	Preclinical	*Parabacteroides goldsteinii* MTS01	Downregulated inflammation through downregulation of COX-2, IL-1β, and TNF-α; decreased pathogenic effect of *H. pylori* virulence factors.

**Table 2:** List of vaccine candidates against *H. pylori* and their potent immune response in animal models.

Authors	Name of Vaccine	Type of Vaccine	Vaccine Delivery	Target	Model Used	Type of Immune Response Elicited
Cen et al. (2021) [52]	*Saccharomyces cerevisiae*-based oral vaccine EBY100/pYD1-UreB, EBY100/pYD1-VacA, or EBY100/pYD1-UreB + EBY100/pYD1-VacA	Vector-based	Oral	Urease and VacA	Mice	Humoral and mucosal immune response.
Wang et al. (2021) [53]	*L. monocytogenes*-based vaccine, a multi-epitope chimeric antigen (MECU) containing multiple B cell epitopes	Vector-based	Oral	5 B-cell epitopes from FlaA, AlpB, SabA, and HpaA	Mice	Elicited high levels of IFN-γ, IL-4, and IL-17 in splenic lymphocytes; increased IgA and IgG.
Xie et al. (2021) [54]	Oral multivalent epitope vaccine	Multivalent epitope	Oral	Three Th cell epitopes and five against B cells	Mice	Increased IFN-γ, IL-4, and IL-17 in lymphocyte supernatants to activate Th1, Th2, and Th17 mixed T-cell immune responses; increased IgA and IgG.
Liu et al. (2019) [55]	Outer-membrane vesicles (OMVs) derived from gerbil-adapted *H. pylori* strain 7.13	Outer membrane vesicle	Oral	Membrane proteins of *H. pylori*	Mice	Th2-biased immunity; increased IgA and IgG.
Peng et al. (2018) [56]	Neutrophil-activating protein A subunit (NapA) and *L. lactis* as vector	Vector-based	Oral	Neutrophil-activating protein A subunit	Mice	Polarized Th17 and Th1 responses; increased IgA and IgG.
Espinosa-Ramos et al. (2019) [57]	*H. pylori* 50–52 kDa immunogen-derived peptide antigen with the sequence Met–Val–Thr–Leu–Ile–Asn–Asn–Glu (MVTLINNE)	Peptide antigen	Subcutaneous	Immunogen synthetic peptide	Mice	Induced thymus lymphocytes and significantly induced IL-6.
Pan et al. (2018) [58]	Multivalent epitope-based vaccine cholera toxin B subunit (CTB)-HUUC with the intramucosal adjuvant CTB and tandem copies of B-cell epitopes	Multivalent epitope	Oral	3 B-cell and 9 T-cell epitopes	Mice	*H. pylori*-specific lymphocyte responses and a mixed CD4^+^ T-cell immune response; increased IgA and IgG.

## Data Availability

Data from this review are available from the corresponding authors upon reasonable request.

## Supplementary Materials

The following supporting information can be downloaded at https://www.mdpi.com/article/10.3390/ph16040552/s1: Table S1. List of natural products from plants described in the studies included in this systematic review.
