# Supplementary material for: The Potential of Alternative Therapies and Vaccine Candidates against Helicobacter pylori
Source: Pharmaceuticals (Basel). 2023 Apr 6;16(4):552. doi: 10.3390/ph16040552 (PMC10141204; doi:10.3390/ph16040552)
Supplement: Supplementary file 1 [file pharmaceuticals-16-00552-s001.zip › pharmaceuticals-2235478-supplementary.pdf]

Table S1: List of natural products from plants from the studies included in this systematic review. N/A: the value or test was not tested from the respective study; MIC: minimal inhibitory concentration; MBC: Minimal bactericidal concentration.

**Authors**	**Name of plant/ compound**	**Fraction/compound with best antibacterial activity**	**Metabolite(s) with anti *H. pylori* activity**	**Assay type**	**MIC**	**MBC**	**Mechanism**
Eftekhari et al. (2021) [22]	*Oliveria decumbens* Vent.	n-hexane	N/A	disc diffusion and agar dilution methods	50 µg/mL	N/A	N/A
Palacios-Espinosa et al. (2021) [23]	*Artemisia ludoviciana*	Estafiatin and eupalitin	Estafiatin and eupalitin	broth microdilution assay	250 µg/mL	15.6 µg/mL (estafiatin) and 31.2 µg/mL (eupalitin)	Anti-urease
Lee et al. (2018) [24]	*Allium hookeri*	Alliin; *Allium hookeri* extract	Alliin	disk agar diffusion method	Allin: 15.6 mm; AHE 100 µg/mL (20.6 mm)	N/A	Anti-urease and gastroprotection
Stenger Moura et al. (2021) [25]	*Mimusops balata* (Sapotaceae) edible fruits	Taxifolin	Taxifolin	broth microdilution assay	625 µg/mL	N/A	Reduced ulcer and anti-oxidant
Lu et al. (2020) [26]	*Zanthoxylum nitidum* (Roxb.) DC.	Root aqueous extract	N/A	N/A	N/A	N/A	Anti-urease
Ngnameko et al. (2020) [27]	*Spathodea campanulata* P. Beauv. (Bignoniaceae)	SB2, SE3 and flavonol kaempferol	SB2, SE3 and flavonol kaempferol	N/A	N/A	N/A	Anti-urease; Anti *H. pylori* adhesins; Anti *H. pylori* CagA

Park et al. (2018) [28]	Hwanglyeonhaedok-tang (HHT) (combination of plants)	Water decoction	N/A	N/A	N/A	N/A	Reduced *H. pylori* colonization and anti-inflammatory
Ngan et al. (2021) [29]	*Hibiscus rosa-sinensis* red flower	ethyl acetate fraction	N/A	broth microdilution assay	0.2-0.25 mg/mL	1.25-1.5 mg/mL	Antibiofilm activity; conversion of bacteria to coccoid form
Park et al. (2019) [30]	Lycopene (L9879, Sigma-Aldrich)	Lycopene	Lycopene	N/A	N/A	N/A	Reduced expression of cellular signalling important in gastric carcinogenesis
Salinas Ibáñez et al. (2021) [31]	*Solanum granuloso-leprosum* (Dunal)	Granulosin I and proteolytic extract (RAP)	Granulosin I	broth microdilution assay	All strains susceptible granulosain I with MIC from 156.25 to 312.5 µg/mL ; RAP with MIC from 312.5 to 625 µg/mL	granulosin I: 312.5 to 625 µg/mL; 625 to 1,250 µg/mL for RAP	Significantly decreased the expression of pathogenic factors: *omp18*, *ureA*, and *flaA*.
Jin et al. (2020) [32]	*Curcuma wenyujin*	n-Butyl alcohol extract	N/A	N/A	N/A	N/A	Suppressed effects induced by *H. pylori* CagA and VacA
Wang et al. (2018) [33]	*Physalis alkekengi* L. var. franchetii (Solanaceae)	Ethyl acetate fraction	N/A	disk diffusion method	500µg/ml	N/A	Reduced inflammation; gastroprotective
Fahmy et al. (2020) [34]	*Erythrina speciosa* (Fabaceae)	Acetate fraction	N/A	broth microdilution assay	31.25 µg/mL	N/A	Gastroprotective and anti inflammatory

Lee et al. (2019) [35]	*Rubus crataegifolius,Gardenia jasminoides* and *Ulmus macrocarpa*	Standardized plant extracts	N/A	N/A	N/A	N/A	Reduced bacterial load and inflammation
Jung et al. (2020) [36]	H-002119-00-001 from β-caryophyllene	H-002119-00-001	H-002119-00-001 from β-caryophyllene	N/A	N/A	N/A	Improved inflammation and decreased bacterial burden
Li et al. (2022) [37]	*Forsythia*	Phillygenin	Phillygenin	broth microdilution assay	16-32 μg/ml	128 μg/ml	Caused ATP leakage in *H. pylori* cells; antibiofilm; downregulated *H. pylori*'s acid resistance, DNA methylation and capacity for drug efflux; improved immune response
Eftekhari et al. (2019) [38]	*Oliveria decumbens*	Essential oils and thymol	Essential oils and thymol	disc diffusion and agar dilution methods	essential oil: 20.4 μg/mL and thymol: 150 μg/mL	N/A	N/A
Brito et al. (2018) [39]	*Spondias mombin L.*	Ethanolic extract	N/A	broth microdilution	256 μg/mL	N/A	N/A
Prazeres et al. (2019) [40]	*L. ferrea*	Dry extract of *L. ferrea* pods	N/A	broth microdilution	512 μg/mL	512 μg/mL	N/A

Wylie et al. (2022) [41]	*Azadirachta indica* A. Juss	Nimbolide	Nimbolide	broth microdilution	1.25–5 µg/mL	2.5–10 µg/mL	N/A
Peng et al. (2022) [42]	*Syzygium aromaticum*	Aqueous extract and 75% hydroalcoholic extract (HE)	N/A	broth microdilution method	160-320 µg/ml for both antibiotic susceptible and resistant *H. pylori*	AE<4MIC while HE 2-16 MIC	altered the morphology and ultrastructure and down-regulated the virulence genes expression of *H. pylori*; combated the abnormal activation of PI3K-Akt and MAPK signaling pathways
Sabry et al. (2022) [43]	*Cordia africana* Lam.	Methyl rosmarinate	Methyl rosmarinate	broth microdilution method	31.25 ± 0.6 µg/mL	N/A	N/A
Almeida et al. (2019) [44]	*Virola elongata*	The stem bark hydroethanolic extract of the plant	N/A	broth microdilution method	No direct activity	No direct activity	gastroprotective
So et al. (2019) [45]	*Ulmus davidiana* var. japonica	Compounds 1, 11, 18, and 20	new chromane derivative, coumarins, and phenolic compounds	broth microdilution method	25 or 50 µM against two strains of *H. pylori* 51 and 43504	N/A	N/A
Sreeja et al. (2018) [46]	*Sphenodesme involucrata* var. paniculata (C.B. Clarke) Munir (Lamiaceae)	Methanol extract	N/A	broth microdilution	100 µg/mL	N/A	N/A

de Cássia Dos Santos et al. (2019) [47]	*Byrsonima intermedia* A. Juss partitions	Partitions of water and ethyl acetate	N/A	broth microdilution assay	Ethyl acetate partition>0.1000 mg/mL and water partition= 0.500 mg/mL	N/A	Gastroprotective
Abdel-Baki et al. (2022) [48]	*Iris confusa*	Polar fractions and iregenin	Ireginin	broth microdilution assay	62.50 μg/mL	N/A	Potential COX2 inhibitor
Kim et al. (2022) [49]	Korean red ginseng extract	Water extract	N/A	N/A	N/A	N/A	Decreased IL-8; antioxidant activity

References (as arranged in Reference section in the paper):

22. Eftekhari, M.; Shams Ardekani, M. R.; Amin, M.; Mansourian, M.; Saeedi, M.; Akbarzadeh, T.; Khanavi, M. Anti-*Helicobacter pylori* compounds from Oliveria decumbens Vent. through urease inhibitory in-vitro and in-silico studies. Iran J Pharm Res **2021**, *20*, 476–489.

23. Palacios-Espinosa, J. F.; Núñez-Aragón, P. N.; Gomez-Chang, E.; Linares, E.; Bye, R.; Romero, I. Anti-*Helicobacter pylori* activity of Artemisia ludoviciana subsp. mexicana and two of its bioactive components, estafiatin and eupatilin. Molecules (Basel) **2021**, *26*, 3654.

24. Lee, H. A.; Hong, S.; Yoo, J. H.; Chung, Y.; Kim, O. Anti-*Helicobacter pylori* activity and inhibition of gastritis by *Allium hookeri* extract. Lab Anim Res **2018**, *34*, 75–79.

25. Stenger Moura, F. C.; Cechinel-Filho, V.; Greco, F. A.; Venzon, L.; Meurer, M. C.; França, T. C. D. S.; Longo, B.; Somensi, L. B.; Mariano, L. N. B.; Cruz, A. B.; Macchiarulo, A.; Schoubben, A.; Ricci, M.; Belle Bresolin, T. M.; da Silva, L. M. (2021). Taxifolin and gastro-adhesive microparticles containing taxifolin promotes gastric healing in vivo, inhibits *Helicobacter pylori* in vitro and proton pump reversibly in silico. Chem Biol Interact **2021**, *339*, 109445.

26. Lu, Q.; Li, C.; Wu, G. Insight into the inhibitory effects of *Zanthoxylum nitidum* against *Helicobacter pylori* urease and jack bean urease: Kinetics and mechanism. J Ethnopharmacol **2020**, *249*, 112419.

27. Ngnameko, C. R.; Marchetti, L.; Zambelli, B.; Quotadamo, A.; Roncarati, D.; Bertelli, D.; Njayou, F. N.; Smith, S. I.; Moundipa, P. F.; Costi, M. P.; Pellati, F. New insights into bioactive compounds from the medicinal plant *Spathodea campanulata* P. Beauv. and their activity against *Helicobacter pylori*. Antibiotics (Basel**)** **2020**, *9*, 258.

28. Park, H. S.; Wijerathne, C. U. B.; Jeong, H. Y.; Seo, C. S.; Ha, H.; Kwun, H. J. Gastroprotective effects of Hwanglyeonhaedok-tang against *Helicobacter pylori*-induced gastric cell injury. J Ethnopharmacol **2018**, *216*, 239–250.

29. Ngan, L. T. M.; Tan, M. T.; Hoang, N. V. M.; Thanh, D. T.; Linh, N. T. T.; Hoa, T. T. H.; Nuong, N. T. M.; Hieu, T. T. Antibacterial activity of Hibiscus rosa-sinensis L. red flower against antibiotic-resistant strains of *Helicobacter pylori* and identification of the flower constituents. Brazil J Med Biol Res **2021,** *54*, e10889.

30. Park, B., Lim, J. W., & Kim, H. Lycopene treatment inhibits activation of Jak1/Stat3 and Wnt/β-catenin signaling and attenuates hyperproliferation in gastric epithelial cells. Nutr Res **2019**, *70*, 70–81.

31. Salinas Ibáñez, Á. G.; Vallés, D., Adaro, M.; Barberis, S., & Vega, A. E. Antimicrobial effect of a proteolytic enzyme from the fruits of *Solanum granuloso-leprosum* (Dunal) against *Helicobacter pylori*. Front Nutr **2021**, *8*, 699955.

32. Jin, H. F.; Dai, J. F.; Meng, L. N.; Lu, B. Curcuma wenyujin Y. H. Chen et C. Ling n-butyl alcohol extract inhibits AGS cell *Helicobacter pylori* CagA+VacA+ promoted invasiveness by down-regulating caudal type homeobox transcription factor and claudin-2 expression. Chin J Integr Med **2020**, *26*, 122–129.

33. Wang, Y.; Wang, S. L.; Zhang, J. Y.; Song, X. N.; Zhang, Z. Y.; Li, J. F.; Li, S. Anti-ulcer and anti-Helicobacter pylori potentials of the ethyl acetate fraction of Physalis alkekengi L. var. franchetii (Solanaceae) in rodent. J Ethnopharmacol **2018**, *211*, 197–206.

34. Fahmy, N. M.; Al-Sayed, E.; Michel, H. E.; El-Shazly, M.; Singab, A. N. B. Gastroprotective effects of Erythrina speciosa (Fabaceae) leaves cultivated in Egypt against ethanol-induced gastric ulcer in rats. J Ethnopharmacol **2020**, *248*, 112297.

35. Lee, Y. I.; Kim, J. S.; Cho, J. S.; Kim, H. K.; Hussain, A. Standardized combined plant extract, RUG-com, reduces bacterial levels and suppresses acute and chronic inflammation in Balb/c mice infected with CagA+ *Helicobacter pylori*. Prev Nutr Food Sci **2019**, *24*, 426–433.

36. Jung, D. H.; Park, M. H.; Kim, C. J.; Lee, J. Y.; Keum, C. Y.; Kim, I. S.; Yun, C. H.; Kim, S. K.; Kim, W. H.; Lee, Y. C. Effect of β-caryophyllene from cloves extract on *Helicobacter pylori* eradication in mouse model. Nutrients **2020**, *12*, 1000.

37. Li, R. J.; Qin, C.; Huang, G. R.; Liao, L. J.; Mo, X. Q.; Huang, Y. Q. Phillygenin inhibits *Helicobacter pylori* by preventing biofilm formation and inducing ATP leakage. Front Microbiol **2022**, *13*, 863624.

38. Eftekhari, M.; Shams Ardekani, M. R.; Amin, M.; Attar, F.; Akbarzadeh, T.; Safavi, M.; Karimpour-Razkenari, E.; Amini, M.; Isman, M.; Khanavi, M. Oliveria decumbens, a bioactive essential oil: chemical composition and biological activities. Iran J Pharm Res **2019**, *18*, 412–421.

39. Brito, S. A.; de Almeida, C. L. F.; de Santana, T. I.; da Silva Oliveira, A. R.; do Nascimento Figueiredo, J. C. B.; Souza, I. T.; de Almeida, L. L.; da Silva, M. V.; Borges, A. S.; de Medeiros, J. W.; da Costa Silva Neto, J.; de Cássia Ribeiro Gonçalves, R.; Kitagawa, R. R.; Sant'Ana, A. E. G.; Rolim, L. A.; de Menezes, I. R. A.; da Silva, T. G.; Caldas, G. F. R.; Wanderley, A. G. Antiulcer activity and potential mechanism of action of the leaves of Spondias mombin L. Oxid Med Cell Longev **2018**, 1731459.

40. Prazeres, L. D. K. T.; Aragão, T. P.; Brito, S. A.; Almeida, C. L. F.; Silva, A. D.; de Paula, M. M. F.; Farias, J. S.; Vieira, L. D.; Damasceno, B. P. G. L.; Rolim, L. A.; Veras, B. O.; Rocha, I. G.; Silva Neto, J. C.; Bittencourt, M. L. F.; Gonçalves, R. C. R.; Kitagawa, R. R.; Wanderley, A. G. Antioxidant and antiulcerogenic activity of the dry extract of pods of Libidibia ferrea Mart. ex Tul. (Fabaceae). Oxid Med Cell Longev **2019**, 1983137.

41. Wylie, M. R.; Windham, I. H.; Blum, F. C.; Wu, H.; Merrell, D. S. In vitro antibacterial activity of nimbolide against *Helicobacter pylori*. J Ethnopharmacol **2022**, *285*, 114828.

42. Peng, C.; Sang, S.; Shen, X.; Zhang, W.; Yan, J.; Chen, P.; Jiang, C.; Yuan, Y.; Zhu, W.; Yao, M. In vitro anti-*Helicobacter pylori* activity of *Syzygium aromaticum* and the preliminary mechanism of action. J Ethnopharmacol **2022**, *288*, 114995.

43. Sabry, M. M.; El-Fishawy, A. M.; El-Rashedy, A. A.; El Gedaily, R. A. Phytochemical investigation of *Cordia africana* Lam. stem bark: molecular simulation approach. Molecules (Basel) **2022**, *27*, 4039.

44. Almeida, G. V. B.; Arunachalam, K.; Balogun, S. O.; Pavan, E.; Ascêncio, S. D.; Soares, I. M.; Zanatta, A. C.; Vilegas, W.; Macho, A.; Oliveira Martins, D. T. Chemical characterization and evaluation of gastric antiulcer properties of the hydroethanolic extract of the stem bark of *Virola elongata* (Benth.) Warb. J Ethnopharmacol **2019**, *231*, 113–124.

45. So, H. M.; Yu, J. S.; Khan, Z.; Subedi, L.; Ko, Y. J.; Lee, I. K.; Park, W. S.; Chung, S. J.; Ahn, M. J.; Kim, S. Y.; Kim, K. H. Chemical constituents of the root bark of *Ulmus davidiana* var. japonica and their potential biological activities. Bioorg Chem **2019**, *91*, 103145.

46. Sreeja, P. S.; Arunachalam, K.; Saikumar, S.; Kasipandi, M.; Dhivya, S.; Murugan, R.; Parimelazhagan, T. Gastroprotective effect and mode of action of methanol extract of *Sphenodesme involucrata* var. paniculata (C.B. Clarke) Munir (Lamiaceae) leaves on experimental gastric ulcer models. Biomed Pharmacother **2018,** *97*, 1109–1118.

47. de Cássia Dos Santos, R.; Bonamin, F., Périco, L. L.; Rodrigues, V. P.; Zanatta, A. C.; Rodrigues, C. M.; Sannomiya, M.; Dos Santos Ramos, M. A.; Bonifácio, B. V.; Bauab, T. M.; Tamashiro, J.; da Rocha, L. R. M.; Vilegas, W.; Hiruma-Lima, C. A. *Byrsonima intermedia* A. Juss partitions promote gastroprotection against peptic ulcers and improve healing through antioxidant and anti-inflammatory activities. Biomed Pharmacother **2019**, *111*, 1112–1123.

48. Abdel-Baki, P. M.; El-Sherei, M. M.; Khaleel, A. E.; Abdel-Aziz, M. M.; Okba, M. M. Irigenin, a novel lead from Iris confusa for management of Helicobacter pylori infection with selective COX-2 and HpIMPDH inhibitory potential. Sci Rep **2022**, *12*, 11457.

49. Kim, H. S.; Lim, J. W.; Kim, H. Korean red ginseng extract inhibits IL-8 expression via Nrf2 activation in *Helicobacter pylori*-infected gastric epithelial cells. Nutrients **2022,** *14*, 1044.
